# Prospective association between phthalate exposure in childhood and liver function in adolescence: the Ewha Birth and Growth Cohort Study

**DOI:** 10.1186/s12940-022-00953-w

**Published:** 2023-01-06

**Authors:** Seonhwa Lee, Hye Ah Lee, Bohyun Park, Hyejin Han, Young Sun Hong, Eun Hee Ha, Hyesook Park

**Affiliations:** 1 Department of Preventive Medicine, College of Medicine, Ewha Womans University, 07804 Seoul, Korea; 2 Center of Public Healthcare, National Medical Center, Seoul, 04564 Korea; 3 Clinical Trial Center, Ewha Womans University Mokdong Hospital, Seoul, 07985 Korea; 4 National Cancer Control Institute, National Cancer Center, Goyang, 10408 Korea; 5 Gangdong Public Healthcare Center, Seoul, 05397 Korea; 6 Department of Internal Medicine, College of Medicine, Ewha Womans University, Seoul, 07804 Korea; 7 Department of Occupational and Environmental Medicine, College of Medicine, Ewha Womans University, Seoul, 07804 Korea; 8 Graduate Program in System Health Science and Engineering, Ewha Womans University, 07804 Seoul, Korea

**Keywords:** Phthalates, Liver function, Children, Cohort, Interaction effect

## Abstract

**Background:**

Phthalate exposure is ubiquitous due to the widespread use of plastic products in daily life, and affects several health outcomes, including metabolic diseases. In this study, we evaluated the effects of phthalate exposure in childhood on liver function in adolescence.

**Methods:**

Among 164 Ewha Birth and Growth Cohort Study participants followed up during two exposure periods (when the children were aged 3–5 and 7–9 years), 126 were followed up at age 10–15 years. To investigate the relationship between phthalate exposure during the two periods and liver enzyme levels (ALT, AST, γ-GTP) in adolescence, differences between groups and the dose–response relationship were analyzed. In addition, we investigated differences in liver enzymes between groups based on the combined exposure levels (high or low) during the two periods. The interaction effect between phthalates and BMI on liver enzyme levels was evaluated, stratified by sex.

**Results:**

In the 3–5 year-old exposure period, ALT levels tended to increase as MECPP levels increased, while γ-GTP levels tended to increase as MiBP, MnBP, and ∑DBP levels increased. In addition, the group exposed to consistently high levels of phthalates at both time points had higher liver enzyme levels compared to the group that had lower exposure. In particular, the interaction effect between some phthalate metabolites and BMI in 3–5 year olds affected AST and γ-GTP levels in adolescence only in girls.

**Conclusions:**

Exposure to phthalates in daily life during childhood affects liver enzyme levels in adolescence. Elevated liver enzyme levels are associated with the development of metabolic syndrome, implying that attention should be paid to phthalate exposure during childhood.

**Supplementary Information:**

The online version contains supplementary material available at 10.1186/s12940-022-00953-w.

## Introduction

Phthalates are plasticizers that are ubiquitous in cosmetics, food packaging, medical supplies, and toys. High-molecular-weight (HMW) phthalates such as di-(2-ethylhexyl) phthalate (DEHP) and butyl benzyl phthalate (BBzP) are mainly used as plasticizers in the manufacture of polyvinyl chloride, building materials, and toys. Low-molecular-weight (LMW) phthalates, including di-butyl phthalate (DBP) and diethyl phthalate (DEP), are primarily used in the manufacture of personal-care products such as perfumes, lotions, and cosmetics [1].

During the use of these products, phthalates can be absorbed into the body through the skin, breathing, and food intake [2]. They can disrupt the endocrine system, causing not only diseases related to sex hormones [3] but also respiratory [4] and metabolic diseases [5] such as diabetes and obesity. In particular, children are easily exposed to phthalates, such as by sucking toys, and exposure per body area may be higher than in adults [6]. In addition, as their immune systems are not well-developed, their exposure to endocrine disruptors in daily life must be carefully monitored. It is important to investigate how these public-health problems affect health. Previous studies have reported that phthalate exposure in children causes not only neurodevelopmental problems [7] such as attention deficit hyperactivity disorder (ADHD) [8] but also metabolic diseases such as hypertension [9] and obesity [10].

The liver plays a major role in primary detoxification of various harmful substances, including phthalates, and increased liver enzyme levels are a predictor of metabolic syndrome [11, 12]. Some studies have suggested that phthalates cause liver damage. In an experimental study, rats exposed to DBP developed oxidative stress and had significantly increased liver enzyme levels [alanine aminotransferase (ALT) and aspartate aminotransferase (AST)] [13]. In a cross-sectional study, urine phthalate metabolite concentrations in American adolescents were significantly related to changes in liver-function-test (LFT) indicators. Previous studies have evaluated the association with exposure to environmental substances using AST, ALT, and gamma-glutamyl transferase (γ-GTP) as biomarkers of liver injury in children [14–16]. A cross-sectional study showed that phthalate exposure had potentially toxic effects on humans and BMI modified the association between phthalate and liver injury in adults [17]. However, prospective research in children is lacking.

In this study, we used data from the Ewha Birth and Growth Cohort Study to determine whether repeated phthalate exposure during childhood (at 3–5 and 7–9 years of age) affected liver enzyme levels at the age of 10–15 years.

## Materials and methods

The study subjects consisted of the hospital-based birth cohort of the Ewha Birth and Growth Cohort Study, which enrolled pregnant women in the second trimester (24–28 weeks) who visited the Department of Obstetrics and Gynecology at Ewha Womans University Mokdong Hospital between 2001 and 2006, and whose offspring were followed-up (n = 940). Follow-up started in 2005 (see details in [18]). Of the 940 cohort members, the follow-up rates of subjects who visited at least once during the follow-up period were 67% and 59%, respectively. Consent was obtained from all participants and their parents through a written consent form, and the protocol was approved by the Institutional Review Board (IRB) of Ewha Womans University Hospital (IRB No. SEUMC 2020–07-016).

Blood and urine samples and body measurements were collected from each participant through follow-up observations in each period, and demographic and socioeconomic information were collected through structured questionnaires. Venous blood was collected from the children in the morning after fasting for 8–12 h using a vacuum tube containing ethylenediaminetetraacetic acid (EDTA). The collected samples were stored at –80 °C in a freezer, and were transported in the frozen state for examination.

In total, 164 participants with measurable phthalate in urine samples participated in the follow-up for two exposure periods (3–5 and 7–9 years of age). Among them, 126 participated in further follow-up examinations at 10–15 years of age, and were included in the analysis.

### Exposure assessment: measurement of urinary phthalate metabolite concentrations

Urinary concentrations of phthalate metabolites in the study subjects were repeatedly measured during both exposure periods (at 3–5 and 7–9 years of age). The measured metabolites were mono-benzyl phthalate (MBzP), mono(2-ethyl-5-carboxypentyl) phthalate (MECPP), mono(2-ethyl-5hydroxyhexyl) phthalate (MEHHP), mono(2-ethylhexyl) phthalate (MEHP), mono(2-ethyl-5-oxohexyl phthalate (MEOHP), monoethyl phthalate (MEP), monoisobutyl phthalate (MiBP), monoisononyl phthalate (MiNP), and mono-n-butyl phthalate (MnBP). ΣDEHP was calculated as the molar sum of MECPP, MEHHP, MEHP, and MEOHP, and ΣDBP was calculated as the molar sum of MnBP and MiBP. The molar sum was calculated by dividing each metabolite concentration by its molar mass and then summing the individual metabolite concentrations (μmol/L).

Stored spot urine samples (≥ 1 mL) were sent to a specialized diagnostic laboratory (Lab Frontier, Korea) to measure phthalate metabolites. After adding 50 µL internal standard solution (200 ng/mL, nine types of phthalate metabolites-^13^C_12_), 1 mL 2 M ammonium acetate buffer, and 20 µL β-glucuronidase, the urine samples were incubated overnight at 37 °C. Thereafter, 4 mL ethyl acetate was added and extracted twice. The extracted samples were gently shaken several times and centrifuged at 4,000 rpm for 15 min to separate the non-polar fat layer and the organic layer. The upper organic layer, which did not contain non-polar fat, was separated and transferred to a glass tube. The extract was evaporated and dried at 40 °C under a gentle flow of nitrogen, and then re-extracted with 100 µL acetonitrile.

The analysis was performed using high-performance liquid chromatography (HPLC). For HPLC tandem mass spectrometry (MS/MS), Agilent 1200 series and Agilent 6430 Triple Quad liquid chromatograph mass spectrometers (Agilent Technologies Inc., Santa Clara, CA, USA) were used. A CAPCELL PAK C18 MG II column (3.0 × 150 mm, 3 μm) (Shiseido Co., Ltd., Tokyo, Japan) was used for chromatographic separation. The mobile phase was carried out at a flow rate of 0.3 mL/min using 0.1% acetic acid in distilled water and 0.1% acetic acid in acetonitrile. MS/MS was analyzed in electrospray negative ion multiple reaction monitoring mode. The accuracy of the analysis was 70–120%, and the coefficients of variation (CVs) were 0.77% (MBzP), 2.27% (MECPP), 1.21% (MEHHP), 2.41% (MEHP), 1.17% (MEOHP), 3.09% (MEP), 0.89% (MiBP), 1.34% (MiNP), and 1.88% (MnBP). In addition, the limits of detection (LODs) were 0.27 μg/L (MBzP), 0.16 μg/L (MECPP), 0.19 μg/L (MEHHP), 0.24 μg/L (MEHP), 0.22 μg/L (MEOHP), 1.0 μg/L (MEP), 0.20 μg/L (MiBP), 0.17 μg/L (MiNP), and 0.26 μg/L (MnBP). To calculate creatinine-adjusted phthalate concentrations, creatinine was measured by the Jaffé method [19], using Olympus AU 680 (Beckman Coulter, Inc., Brea, CA, USA) equipment.

### Health outcomes: measurement of blood liver enzyme concentrations

The stored blood samples (≥ 1 mL) of the 126 children who completed the follow-up at age 10–15 years were sent to a specialized diagnostic laboratory (Seegene Medical Institute, Korea) for analysis. AST, ALT, and γ-GTP were measured as indicators of liver enzyme levels in the blood. These were measured using Cobas 8000 C702 (Roche, Germany) equipment by the enzymatic method. ASTL, ALTL, and GGT Gen.2 (Roche) reagents were used for AST, ALT, and γ-GTP, respectively. In all of the measured indicators, the CV of accuracy was < 5%.

### Covariates

Covariates were considered based on a literature review and directed acyclic graph (DAG) (Figure S1); variables associated with liver enzymes at least once based on *p* < 0.2 were considered for analysis. All analyses were performed after adjusting for confounding factors such as sex, age, BMI, mother’s education level, household income level, secondhand smoke, and physical inactivity. The BMI was calculated using body measurements obtained during follow-up as weight (kg) divided by the square of height (m^2^). Height and weight were measured in 0.1-cm and 0.1-kg units using an automatic height and weight measurement system (DS-102; Dong Sahn Jenix Co., Ltd., Seoul, Korea), without shoes and in light clothing.

The mothers’ education levels were divided into two categories (graduated from high school or lower and graduated from college or higher) based on the questionnaire responses (graduated from elementary/middle school, high school, college, or above graduate school). Household income was divided into three categories (˂3 million won, 3–5 million won, and ≥ 5 million won) based on the questionnaire responses about monthly average household income (˂1 million won, 1–2 million won, 2–3 million won, 3–5 million won, or ≥ 5 million won). A questionnaire item was used to assess each child’s exposure to secondhand smoke on follow-up at the age of 10–15 years. The level of physical inactivity at age 10–15 years was divided into three categories (< 1 h, ≥ 1 h, and ≥ 2 h) based on the questionnaire items regarding the average time spent sitting in leisure activities such as watching TV and playing games (< 1 h, ≥ 1 h, ≥ 2 h, and almost none).

### Statistical analysis

When the concentration of phthalate metabolites in urine (μg/L) was less than the LOD, LOD/√2 was used for analysis [20]. When a subject visited more than once at the age of 10–15 years, liver enzyme levels at the time of the earliest visit were preferentially used for analysis. Continuous and normally distributed data are presented as means and standard deviations, and categorical data are presented as frequencies and percentages (%).

Analysis of covariance (ANCOVA) was performed to determine the differences in liver enzyme levels (ALT, AST, and γ-GTP) between groups by dividing phthalate exposure into tertiles at two time points (when the children were 3–5 and 7–9 years of age). To assess the dose–response relationship, a trend analysis was performed based on exposure levels by applying an ordinal variable. We used a restrictive cubic spline (RCS) model to evaluate nonlinear relationships. In addition, a sensitivity analysis was performed to exclude the effects of BMI as a mediator, on determining the results.

Exposure levels were redefined for high and low exposure groups based on the medians for the 3–5 and 7–9 years age groups, to assess the differences between exposure levels at the two exposure periods: low exposure levels in both periods (LL); high at 3–5 years, but low at 7–9 years (HL); low at 3–5 years, but high at 7–9 years (LH); and high in both periods (HH). This was to assess whether there were any differences in the mean liver enzyme levels between groups. If a difference was significant, Bonferroni’s post hoc test was performed.

The relationships between phthalate metabolites and liver enzymes were evaluated using linear regression. Furthermore, the interaction effects of BMI with urinary phthalate according to sex were evaluated using linear regression.

All statistical analyses were performed using SAS ver. 9.4 (SAS Institute, Cary, NC, USA) and R Statistical Software (ver. 3.6.2; R Core Team 2019). Statistical significance was tested based on α = 0.05 using two-sided tests.

## Results

In a previous study that measured phthalate exposure levels using urine samples from the same subjects as in this study, the geometric means of ΣDEHP and ΣDBP at 3–5 years of age were 377.0 μg/g creatinine and 239.4 μg/g creatinine, respectively. The geometric means of ΣDEHP and ΣDBP at 7–9 years of age were 229.0 μg/g creatinine and 154.7 μg/g creatinine, respectively, and the levels were higher in the 3–5 years age group than in the 7–9 years group [21]. In addition, when the molar sum was calculated by dividing by the molar mass of each metabolic concentration, the geometric means of ΣDEHP and ΣDBP at 3–5 years of age were 1.27 and 1.08 μmol/g creatinine, respectively. The respective geometric means of ΣDEHP and ΣDBP at 7–9 years of age were 0.83 and 0.75 μmol/g creatinine.

There were no differences in basic characteristics (sex, BMI, mother’s education levels, and monthly household income) during childhood (at 3–5 and 7–9 years of age) between those included and not included in the analysis. The distribution of liver enzyme levels in the study population was almost within the normal range, and the children and their mothers never had liver disease or hepatitis.

Table 1 summarizes the descriptive characteristics of the study subjects. Among 164 children whose exposure to phthalates was measured at the ages of 3–5 and 7–9 years, the numbers of boys and girls were similar (50.6% vs. 49.4%). The mothers’ education levels included a high proportion of college graduates or higher. The average BMI was 15.8 and 17.0 kg/m^2^ for the 3–5 and 7–9 years age groups, respectively. As for monthly household income, those belonging to the middle group accounted for the highest proportion. Of the 164 children, 126 underwent liver enzyme measurements at the age of 10–15 years. The mean AST, ALT, and γ-GTP levels were 18.6, 12.8, and 13.9 IU/L, respectively. In addition, most children were not exposed to secondhand smoke. The time spent sitting in leisure activities, which is an indicator of physical inactivity, was the lowest proportion in within 1 h.

**Table 1:** Descriptive characteristics of the study subjects

Characteristic	n	Mean ± SD or %
Sex		
Boys	83	50.6%
Girls	81	49.4%
Mother’s education level		
Low (graduated of high school or lower)	35	21.3%
High (graduated of college or higher)	129	78.7%
3–5 years of age		
BMI (kg/m^2^)	164	15.8 ± 1.6
Normal	142	86.6%
Overweight ^a^	22	13.4%
Monthly household income		
Low (< 3 million won)	41	26.6%
Middle (< 5 million)	65	42.2%
High (≥ 5 million)	48	31.2%
7–9 years of age		
BMI (kg/m^2^)	163	17.0 ± 2.4
Normal	143	87.7%
Overweight^a^	20	12.3%
Monthly household income		
Low (< 3 million won)	26	16.1%
Middle (< 5 million)	69	42.9%
High (≥ 5 million)	66	41.0%
10–15 years of age		
Liver enzymes		
AST (IU/L)	126	18.6 ± 6.1
ALT (IU/L)	124	12.8 ± 8.8
γ-GTP (IU/L)	125	13.9 ± 6.7
Second-hand smoke exposure		
No	105	93.8%
Yes	7	6.3%
Physical inactivity level (time spent sitting in leisure time)		
Within 1 h	30	26.3%
Over 1 h	44	38.6%
Over 2 h	40	35.1%

*AST* Aspartate aminotransferase, *ALT* Alanine aminotransferase, *γ-GTP* Gamma-glutamyl transferase, *BMI* Body Mass Index

^a^Overweight was defined as ≥ 85^th^ percentile using 2017 Korean National Growth Charts

Table 2 shows the urinary phthalate levels, divided into tertiles to evaluate whether there was a difference in enzyme levels at 10–15 years of age among the three groups. At 3–5 years of age, after adjusting for potential confounders, there was a statistically significant difference between tertiles in mean AST and ALT levels of some HMW phthalate metabolites (i.e., ∑DEHP) and in mean γ-GTP levels between some LMW phthalates (MiBP, MnBP, and ∑DBP). In the 7–9 years age group, the differences between tertiles in mean AST and ALT levels for LMW phthalates and in mean γ-GTP levels were significant only for MiNP. To determine the dose–response relationship, we evaluated whether higher urine phthalate exposure levels were associated with higher liver enzyme levels. At 3–5 years of age, γ-GTP levels tended to increase as exposure to LMW phthalates (MiBP, MnBP, and ∑DBP) increased, while at 7–9 years of age, AST and ALT levels tended to increase. There was a tendency for ALT levels to increase as exposure to MECPP increased at the age of 3–5 years (*p*_*trend*_ < 0.05). We also evaluated nonlinear relationships using the RCS model, and U-shaped nonlinear relationships of ΣDEHP at 3–5 years with AST at 10–15 years of age were observed. MEHP at 3–5 years of age showed non-linear relationships with all three liver enzymes. However, phthalate exposure at 7–9 years of age did not show a clear non-linear relationship with liver enzymes (Figures S2 to S7). In addition, the effects of size and direction were similar when sensitivity analysis was performed, excluding BMI, which can affect liver enzyme levels (Table S1).

**Table 2:** Differences in liver enzymes according to tertiles of urinary phthalate levels

	AST (IU/L)				ALT (IU/L)				γ-GTP (IU/L)			
	Lsmeans	SE	*p*	*p*_*trend*_	Lsmeans	SE	*p*	*p*_*trend*_	Lsmeans	SE	*p*	*p*_*trend*_
3–5 years of age												
MBzP ^a^	(*n* = 103)				(*n* = 101)				(*n* = 102)			
T1	16.75	1.44	0.093	0.056	10.75	1.47	0.371	0.141	13.53	1.71	0.106	0.193
T2	16.72	1.32			11.29	1.34			12.19	1.58		
T3	19.15	1.37			12.56	1.40			15.40	1.63		
MECPP ^a^	(*n* = 104)				(*n* = 102)				(*n* = 103)			
T1	16.65	1.50	0.091	0.051	9.27	1.48	0.026	0.009	11.88	1.82	0.339	0.167
T2	16.50	1.33			11.29	1.31			13.81	1.60		
T3	18.96	1.32			12.75	1.31			13.99	1.59		
MEHHP ^a^	(*n* = 104)				(*n* = 102)				(*n* = 103)			
T1	18.30	1.51	0.210	0.850	11.28	1.52	0.226	0.215	12.23	1.80	0.232	0.083
T2	16.38	1.34			10.54	1.35			12.89	1.60		
T3	18.37	1.33			12.71	1.34			14.77	1.58		
MEHP ^a^	(*n* = 104)				(*n* = 102)				(*n* = 103)			
T1	18.78	1.50	0.048	0.799	12.16	1.54	0.419	0.918	15.63	1.80	0.115	0.296
T2	15.85	1.36			10.62	1.40			12.32	1.64		
T3	18.28	1.27			12.05	1.30			13.73	1.52		
MEOHP ^a^	(*n* = 104)				(*n* = 102)				(*n* = 103)			
T1	17.28	1.50	0.143	0.235	11.11	1.52	0.237	0.207	12.56	1.81	0.507	0.225
T2	16.20	1.37			10.45	1.39			13.13	1.65		
T3	18.74	1.31			12.63	1.32			14.36	1.58		
MEP ^a^	(*n* = 104)				(*n* = 102)				(*n* = 103)			
T1	17.01	1.43	0.731	0.376	10.38	1.42	0.316	0.126	11.95	1.68	0.192	0.056
T2	17.35	1.43			11.65	1.43			13.37	1.67		
T3	18.05	1.35			12.35	1.34			14.83	1.58		
MiBP ^a^	(*n* = 104)				(*n* = 102)				(*n* = 103)			
T1	18.09	1.49	0.066	0.191	10.94	1.49	0.082	0.056	12.71	1.76	0.036	0.021
T2	16.63	1.24			11.10	1.24			12.94	1.46		
T3	19.66	1.46			13.53	1.47			16.20	1.72		
MiNP ^a^	(*n* = 104)				(*n* = 102)				(*n* = 103)			
T1	16.58	1.44	0.470	0.344	11.08	1.45	0.837	0.594	13.85	1.73	0.949	0.766
T2	18.08	1.41			11.77	1.43			13.72	1.69		
T3	17.77	1.34			11.75	1.35			13.36	1.61		
MnBP ^a^	(*n* = 104)				(*n* = 102)				(*n* = 103)			
T1	17.98	1.45	0.095	0.351	11.10	1.47	0.194	0.134	12.71	1.70	0.021	0.022
T2	16.34	1.30			10.89	1.31			12.36	1.52		
T3	19.10	1.39			13.05	1.41			16.17	1.63		
∑DEHP ^a^	(*n* = 104)				(*n* = 102)				(*n* = 103)			
T1	17.64	1.48	0.041	0.280	11.34	1.47	0.010	0.113	12.61	1.80	0.227	0.133
T2	15.55	1.39			9.25	1.37			12.34	1.68		
T3	18.82	1.27			13.21	1.26			14.76	1.54		
∑DBP ^a^	(*n* = 104)				(*n* = 102)				(*n* = 103)			
T1	18.28	1.51	0.117	0.373	11.11	1.52	0.142	0.089	13.13	1.76	0.018	0.022
T2	16.71	1.24			11.10	1.24			12.65	1.44		
T3	19.44	1.49			13.41	1.50			16.68	1.73		
7–9 years of age												
MBzP ^b^	(*n* = 110)				(*n* = 108)				(*n* = 109)			
T1	17.16	1.32	0.839	0.477	11.90	1.39	0.803	0.957	13.71	1.73	0.704	0.640
T2	17.45	1.22			11.04	1.27			12.93	1.59		
T3	17.84	1.28			11.80	1.35			14.32	1.67		
MECPP ^b^	(*n* = 110)				(*n* = 108)				(*n* = 109)			
T1	16.99	1.35	0.386	0.192	10.22	1.41	0.296	0.136	11.18	1.74	0.111	0.126
T2	17.16	1.16			11.62	1.21			14.34	1.49		
T3	18.51	1.28			12.24	1.34			14.03	1.65		
MEHHP ^b^	(*n* = 110)				(*n* = 108)				(*n* = 109)			
T1	17.12	1.29	0.058	0.074	10.87	1.38	0.371	0.201	12.02	1.72	0.269	0.119
T2	16.49	1.15			11.06	1.23			13.56	1.53		
T3	19.19	1.23			12.50	1.32			14.57	1.64		
MEHP ^b^	(*n* = 110)				(*n* = 108)				(*n* = 109)			
T1	17.31	1.41	0.794	0.494	10.54	1.48	0.476	0.222	10.99	1.81	0.102	0.063
T2	17.33	1.12			11.47	1.17			14.00	1.44		
T3	18.06	1.36			12.14	1.42			14.07	1.74		
MEOHP ^b^	(*n* = 110)				(*n* = 108)				(*n* = 109)			
T1	16.54	1.31	0.294	0.104	10.46	1.38	0.444	0.216	11.78	1.71	0.203	0.364
T2	17.32	1.15			11.57	1.21			14.52	1.50		
T3	18.47	1.26			12.12	1.33			13.33	1.64		
MEP ^b^	(*n* = 110)				(*n* = 108)				(*n* = 109)			
T1	16.71	1.20	0.078	0.078	10.73	1.28	0.197	0.145	12.74	1.61	0.646	0.333
T2	16.14	1.33			10.44	1.41			13.70	1.78		
T3	18.71	1.17			12.55	1.24			14.20	1.57		
MiBP ^b^	(*n* = 110)				(*n* = 108)				(*n* = 109)			
T1	15.60	1.22	0.025	0.007	9.82	1.29	0.084	0.046	12.39	1.63	0.201	0.551
T2	18.11	1.32			12.39	1.41			15.47	1.76		
T3	18.87	1.17			12.55	1.24			13.59	1.56		
MiNP ^b^	(*n* = 110)				(*n* = 108)				(*n* = 109)			
T1	16.82	1.26	0.493	0.477	10.94	1.32	0.620	0.362	11.51	1.61	0.047	0.103
T2	18.24	1.29			11.10	1.37			15.29	1.65		
T3	17.60	1.20			12.09	1.26			14.13	1.52		
MnBP ^b^	(*n* = 110)				(*n* = 108)				(*n* = 109)			
T1	16.49	1.23	0.100	0.026	10.53	1.29	0.086	0.037	12.28	1.64	0.391	0.313
T2	17.25	1.22			11.07	1.28			14.40	1.62		
T3	18.96	1.23			13.11	1.29			14.01	1.63		
∑DEHP ^b^	(*n* = 110)				(*n* = 108)				(*n* = 109)			
T1	17.16	1.37	0.674	0.362	10.43	1.43	0.463	0.257	11.21	1.76	0.104	0.437
T2	17.41	1.11			11.71	1.17			14.59	1.43		
T3	18.22	1.37			11.99	1.44			12.69	1.77		
∑DBP ^b^	(*n* = 110)				(*n* = 108)				(*n* = 109)			
T1	16.10	1.25	0.011	0.002	10.30	1.31	0.011	0.005	12.26	1.70	0.433	0.214
T2	17.00	1.16			10.72	1.22			13.92	1.57		
T3	19.40	1.20			13.57	1.25			14.19	1.62		

Tertiles of phthalate at 3-5 years of age: MBzP(μg/g creatinine): T1: ≤ 7.26; T3: > 16.80; MECPP(μg/g creatinine): T1: ≤ 87.97; T3: > 152.11; MEHHP(μg/g creatinine): T1: ≤ 98.76; T3: > 188.49; MEHP(μg/g creatinine): T1: ≤ 17.17; T3: > 31.69; MEOHP(μg/g creatinine): T1: ≤ 63.56; T3: > 113.67; MEP(μg/g creatinine): T1: ≤ 16.81; T3: > 36.02; MiBP(μg/g creatinine): T1: ≤ 65.73; T3: > 121.24; MiNP(μg/g creatinine): T1: ≤ 3.94; T3: > 6.36; MnBP(μg/g creatinine): T1: ≤ 102.16; T3: > 182.81; ∑DEHP(μmol/g creatinine): T1: ≤ 0.97; T3: > 1.60; ∑DBP(μmol/g creatinine): T1: ≤ 0.77; T3: > 1.35

Tertiles of phthalate at 7-9 years of age: MBzP(μg/g creatinine): T1: ≤ 5.97; T3: > 13.19; MECPP(μg/g creatinine): T1: ≤ 55.91; T3: > 100.57; MEHHP(μg/g creatinine): T1: ≤ 69.00; T3: > 110.70; MEHP(μg/g creatinine): T1: ≤ 12.93; T3: > 24.57; MEOHP(μg/g creatinine): T1: ≤ 39.49; T3: > 66.81; MEP(μg/g creatinine): T1: ≤ 10.35; T3: > 18.86; MiBP(μg/g creatinine): T1: ≤ 49.64; T3: > 81.93; MiNP(μg/g creatinine): T1: ≤ 3.41; T3: > 5.65; MnBP(μg/g creatinine): T1: ≤ 76.64; T3: > 134.57; ∑DEHP(μmol/g creatinine): T1: ≤ 0.64; T3: > 1.04; ∑DBP(μmol/g creatinine): T1: ≤ 0.55; T3: > 0.99

*MBzP* Mono-benzyl phthalate, *MECPP* Mono(2-ethyl-5-carboxypentyl) phthalate, *MEHHP* Mono(2-ethyl-5hydroxyhexyl) phthalate, *MEHP* Mono(2-ethylhexyl) phthalate, *MEOHP* Mono(2-ethyl-5-oxohexyl) phthalate, *MEP* Monoethyl phthalate, *MiBP* Monoisobutyl phthalate, *MiNP* Monoisononyl phthalate, *MnBP* Mono-n-butyl phthalate, *ΣDEHP* Di(2-ethylhexyl) phthalate = MECPP, MEHHP, MEHP and MEOHP, ∑DBP (di-n-butyl phthalate) = MiBP and MnBP

*AST* Aspartate aminotransferase, *ALT* Alanine aminotransferase, *γ-GTP* Gamma-glutamyl transferase

^a^Adjusted for age, sex, BMI, mother’s education level, monthly household income at 3–5 years of age, second-hand smoke exposure, and physical inactivity level at 10–15 years of age

^b^Adjusted for age, sex, BMI, mother’s education level, monthly household income at 7–9 years of age, second-hand smoke exposure, and physical inactivity level at 10–15 years of age

**Fig. 1:**
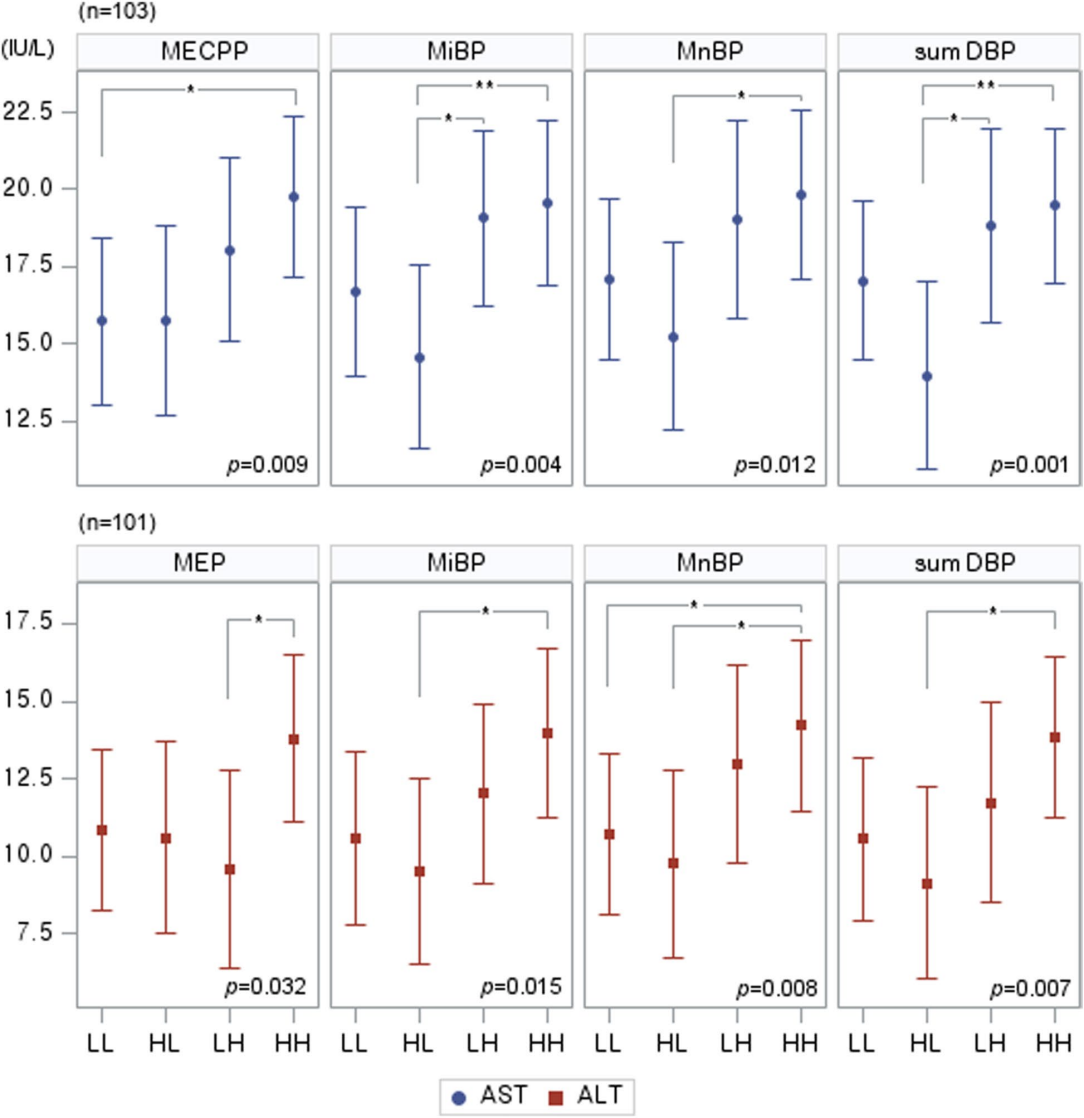
Comparison of liver enzyme levels between the four exposure-level (low or high) groups during each exposure period. LL: low concentrations of phthalate metabolites at the ages of 3–5 years and 7–9 years. HL: high concentrations of phthalate metabolites at the age of 3–5 years, but low concentrations at 7–9 years. LH: low concentrations of phthalate metabolites at the age of 3–5 years, but high concentrations at 7–9 years. HH: high concentrations of phthalate metabolites at the ages of 3–5 years and 7–9 years. MECPP [mono(2-ethyl-5-carboxypentyl) phthalate], MEP (monoethyl phthalate), MiBP (monoisobutyl phthalate), MnBP (mono-n-butyl phthalate), ∑DBP (di-n-butyl phthalate) = MiBP and MnBP. AST; aspartate aminotransferase, ALT; alanine aminotransferase, γ-GTP; gamma-glutamyl transferase. ^*^ *p* < 0.05, ^**^ *p* < 0.01. Adjusted for age, sex, BMI, mother’s education level, monthly household income at 3–5 years of age, second-hand smoke exposure, and physical inactivity level at 10–15 years of age

Figure 1 shows the difference in enzyme levels between the combined groups based on exposure levels in each exposure period. There were significant differences between the combined groups in the mean AST and ALT levels of some phthalates. In the post hoc test, the mean difference in AST and ALT levels between the combined groups was found mainly in LMW phthalates. For HMW phthalates, only the mean differences in AST between the MECPP combined groups were significant (Bonferroni-adjusted *p* < 0.05). In addition, liver enzyme levels were higher in groups with high exposure than in those with low-exposure at 7–9 years of age. In terms of additive interactions, only MEP at both time points had a significant interaction effect on ALT levels (*p*_interaction_ = 0.038).

**Fig. 2:**
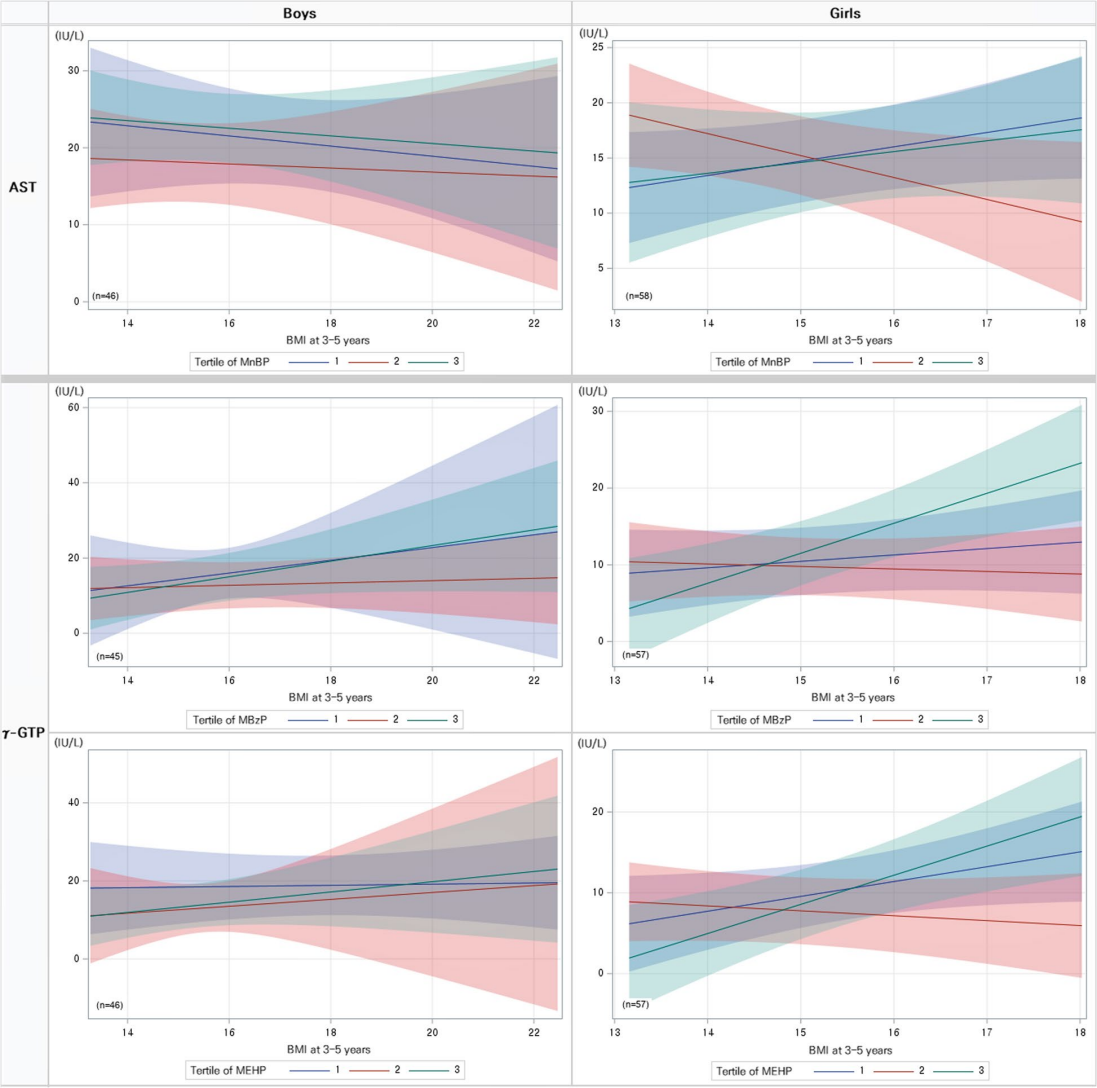
The effect of the interaction between phthalate levels and BMI at 3–5 years of age on liver enzyme levels at 10–15 years of age based on sex. MBzP (mono-benzyl phthalate), MEHP [mono(2-ethylhexyl) phthalate], MnBP (mono-n-butyl phthalate). AST; aspartate aminotransferase, γ-GTP; gamma-glutamyl transferase. Tertiles of Phthalate at 3–5 years of age: MBzP(μg/g creatinine): T1: ≤ 7.26; T3: > 16.80; MEHP(μg/g creatinine): T1: ≤ 17.17; T3: > 31.69;; MnBP(μg/g creatinine): T1: ≤ 102.16; T3: > 182.81. *Adjusted for age, sex, BMI, mother’s education level, monthly household income at 3–5 years of age, second-hand smoke exposure, and physical inactivity level at 10–15 years of age

We evaluated whether BMI acted as an effect modifier. MiBP and BMI at the age of 7–9 years had an interaction effect on AST (*p*_interaction_ = 0.036). In addition, MBzP and BMI at the age of 3–5 years had an interaction effect on γ-GTP (*p*_interaction_ = 0.053). Meanwhile, based on sex, there was an interaction effect only in girls aged 3–5 years (Fig. 2). The interaction effect of LMW phthalate (MnBP) and BMI on AST was significant (*p*_interaction_ = 0.041), and that of HMW phthalate (MBzP and MEHP) and BMI on γ-GTP (MBzP, *p*_interaction_ = 0.021; MEHP, *p*_interaction_ = 0.010) was significant. This can be interpreted as an additional interaction depending on the statistical analysis method used [22].

## Discussion

We investigated mean differences and the dose–response relationship between liver enzymes and phthalate tertiles. At the age of 3–5 years, ALT levels increased with the exposure to MECPP, an HMW phthalate, while γ-GTP levels tended to increase with increasing exposure to MiBP, MnBP, and ΣDBP, which are LMW phthalates. Meanwhile, AST and ALT levels increased at the age of 7–9 years with increasing exposure to LMW phthalates. We combined the phthalate levels of the two exposure periods to demonstrate that consistently high levels of phthalate exposure increased liver enzyme levels, and that this phenomenon was more prominent with LMW phthalates. The liver enzyme levels tended to be higher when the exposure was higher at 7–9 years than at 3–5 years of age (HL vs. HH), excluding MEP. Therefore, exposure to phthalate metabolites at 7–9 years seems to have had a greater effect on liver function than exposure at 3–5 years of age. We found that the effects of exposure to MBzP phthalate at ages 3–5 for γ-GTP at 10–15 years of age differed depending on the BMI at the same time point. After stratification by sex, this was evident only in girls. In addition, some phthalate metabolites and BMI at 3–5 years of age have been found to act as effect modifiers on teenage AST and γ-GTP levels only in girls. For the AST level, MiBP at the age of 7–9 also had an interaction effect with BMI at the same time point, but it did not show a significant effect when stratified by sex.

In a previous study that measured phthalate metabolite concentrations in the same subjects, the phthalate exposure levels were higher at the age of 3–5 years than at the age of 7–9 years [21]. Other studies have also reported that levels of exposure to phthalate metabolites were inversely related to age [23, 24].

Several studies have reported an association between phthalate exposure and metabolic diseases [6], such as obesity [25, 26], hyperlipidemia, and insulin resistance [21]. We considered increased liver enzyme levels to be a possible predictor of the onset of metabolic syndrome, and also reviewed their association with liver-related diseases. In a cross-sectional study of the effects of various levels of phthalate exposure on the liver that included 6,046 adults aged ≥ 20 years in the United States, there was a statistically significant positive relationship between ΣDEHP and changes in ALT, γ-GTP, and ALP levels [17]. A cross-sectional study was conducted to evaluate the association between urinary phthalate levels and liver function in American adolescents aged 12–19 years. Linear regression demonstrated a null association between phthalate metabolites and ALT, AST, γ-GTP, ALP, and ALT/AST levels [27]. In an experimental study of the effects of ΣDBP on liver tissues in three generations of rats, liver enzyme levels (ALT and AST) were significantly increased in rats exposed to DBP [13]. In this study, the effect of phthalates on liver enzyme levels differed slightly according to the difference in exposure levels between the ages of 3–5 and 7–9 years of age. We found that, among HMW phthalates, only MECPP tended to increase ALT levels at the age of 3–5 years. The degree of exposure may differ between countries due to differences in government regulations and lifestyles, and associations with health effects may also differ. Non-alcoholic fatty liver disease (NAFLD) is a condition that can occur even in children and adolescents. An association between phthalates and NAFLD has been reported in adults [28, 29]. A rodent study of the effects of endocrine disruptors on NAFLD reported that early-life DEHP exposure may have long-term consequences for hepatic metabolic function, and exposure to endocrine-disrupting chemicals may be more detrimental in early life than in adulthood [30]. This study did not consider phthalate co-exposure. In a previous cross-sectional study of 12- to 19-year-olds, there was no association between each phthalate metabolite and liver function, but Bayesian kernel machine regression analysis of a phthalate metabolite mixture had significant positive associations with ALT, AST, and γ-GTP [27]. However, insufficient studies have evaluated the relationship with liver function, particularly prospective studies of children and adolescents.

The effects of phthalate metabolites on liver enzyme levels in children and the possible interaction with BMI remain unclear. A cross-sectional study in adults of the effects of phthalates on liver injury found that the association between only MBP and γ-GTP was significantly modified by BMI (*p*_interaction_ = 0.006) [17]. A study of the effects of phthalates on recurrent breast cancer and possible interaction with BMI found that the association between MEOHP and recurrent breast cancer was significantly modified by BMI (*p*_interaction_ = 0.042) [31]. Further investigation of the BMI interaction and the relationship to the gender difference is needed.

The biological action of phthalates is similar to that of peroxisome proliferators, which activate a different subtype of peroxisome proliferator-activated receptors (PPARs), and the liver is a major target organ for their pleiotropic action [17]. Previous studies on rodents have reported that phthalates cause liver toxicity through PPAR activation [32]. An experimental study on rats found oxidative stress and liver damage in rats exposed to ΣDBP that was caused by changes in transaminase activity [13].

The strength of this study was that birth cohort data were used to assess prospectively the causal relationship between daily phthalate exposure in childhood and liver enzyme levels in adolescence. In addition, degrees of exposure were repeatedly measured to evaluate the effects of exposure level at two ages on liver function. This study addresses the lack of previous prospective studies examining the association between repeatedly measured phthalate exposure levels and liver enzyme concentrations in children. In addition, we investigated the effects of phthalate exposure in childhood on liver function, and found a dose–response relationship in which liver enzyme levels increased as exposure to some metabolites increased. Finally, because this study was performed in children, possible effects of risk factors such as chronic diseases, alcohol use, and smoking, which can all negatively affect liver function, were excluded.

A limitation of this study was that residual confounding variables may still have been present. However, because various confounding factors were considered, this effect is considered to have been small, and similar results were obtained even after excluding obesity. Second, because exposure levels were assessed on the basis of measurements of samples collected once at each follow-up period, there may have been measurement errors. Third, phthalates have a short half-life, but it can be assumed that children are exposed to phthalates continuously in their daily lives [6]. Finally, because this study had a small sample, the statistical power was low in some analyses when stratified by sex.

## Conclusion

Exposure to phthalates in daily life during childhood had a dose–response relationship with liver enzyme levels in adolescence. For some phthalate metabolites, levels in the group with relatively high exposure levels had a negative effect on liver enzyme levels compared to the group with low exposure. In particular, interactions between some phthalate metabolites and BMI affected AST and γ-GTP levels in girls, but not in boys. An increase in liver enzyme levels is associated with the onset of metabolic syndrome, implying that daily-life phthalate exposure during childhood requires attention.

## Supplementary Information

**Additional file 1:** **Table S1.** Analysis of BMI sensitivity for differences in liver enzymes according to tertiles of urinary phthalate levels. **Figure S1.** A directed acyclic graph (DAG) depicting the causal relationship between exposure to phthalate metabolites and liver function. **Figure S2.** Non-linear relationship of aspartate aminotransferase(AST) with urinary phthalate levels at 3-5 years of age. **Figure S3.** Non-linear relationship of alanine aminotransferase(ALT) with urinary phthalate levels at 3-5 years of age. **Figure S4.** Non-linear relationship of gamma-glutamyl transferase(γ-GTP) with urinary phthalate levels at 3-5 years of age. **Figure S5.** Non-linear relationship of aspartate aminotransferase(AST) with urinary phthalate levels at 7-9 years of age. **Figure S6.** Non-linear relationship of alanine aminotransferase(ALT) with urinary phthalate levels at 7-9 years of age. **Figure S7.** Non-linear relationship of gamma-glutamyl transferase(γ-GTP) with urinary phthalate levels at 7-9 years of age.

## Data Availability

The datasets used and/or analyzed during the current study are available from the corresponding author on reasonable request.

## Abbreviations

HMW: High-molecular-weight
LMW: Low-molecular-weight
MBzP: Mono-benzyl phthalate
MECPP: Mono(2-ethyl-5-carboxypentyl) phthalate
MEHHP: Mono(2-ethyl-5hydroxyhexyl) phthalate
MEHP: Mono(2-ethylhexyl) phthalate
MEOHP: Mono(2-ethyl-5-oxohexyl phthalate
MEP: Monoethyl phthalate
MiBP: Monoisobutyl phthalate
MiNP: Monoisononyl phthalate
MnBP: Mono-n-butyl phthalate
DEHP: Di-(2-ethylhexyl) phthalate
DBP: Di-butyl phthalate
LFT: Liver-function-test
ALT: Alanine aminotransferase
AST: Aspartate aminotransferase
γ-GTP: Gamma-glutamyl transferase
LL: Low concentrations of phthalate metabolites at the ages of 3–5 years and 7–9 years
HL: High concentrations of phthalate metabolites at the age of 3–5 years, but low concentrations at 7–9 years
LH: Low concentrations of phthalate metabolites at the age of 3–5 years, but high concentrations at 7–9 years
HH: High concentrations of phthalate metabolites at the ages of 3–5 years and 7–9 years
EDTA: Ethylenediaminetetraacetic acid
HPLC: High-performance liquid chromatography
MS/MS: Tandem mass spectrometry
DAG: Directed acyclic graph
CVs: Coefficients of variation
ANCOVA: Analysis of covariance
RCS: Restrictive cubic spline
BMI: Body mass index
ADHD: Attention deficit hyperactivity disorder
NAFLD: Non-alcoholic fatty liver disease
PPARs: Peroxisome proliferator-activated receptors
