# Supplementary material for: Prospective association between phthalate exposure in childhood and liver function in adolescence: the Ewha Birth and Growth Cohort Study
Source: Environ Health. 2023 Jan 6;22:3. doi: 10.1186/s12940-022-00953-w (PMC9817355; doi:10.1186/s12940-022-00953-w)
Supplement: Supplementary file 1 — Additional file 1: Table S1. Analysis of BMI sensitivity for differences in liver enzymes according to tertiles of urinary phthalate levels. Figure S1. A directed acyclic graph (DAG) depicting the causal relationship between exposure to phthalate metabolites and liver function. Figure S2. Non-linear relationship of aspartate aminotransferase(AST) with urinary phthalate levels at 3-5 years of age. Figure S3. Non-linear relationship of alanine aminotransferase(ALT) with urinary phthalate levels at 3-5 years of age. Figure S4. Non-linear relationship of gamma-glutamyl transferase(γ-GTP) with urinary phthalate levels at 3-5 years of age. Figure S5. Non-linear relationship of aspartate aminotransferase(AST) with urinary phthalate levels at 7-9 years of age. Figure S6. Non-linear relationship of alanine aminotransferase(ALT) with urinary phthalate levels at 7-9 years of age. Figure S7. Non-linear relationship of gamma-glutamyl transferase(γ-GTP) with urinary phthalate levels at 7-9 years of age. [file 12940_2022_953_MOESM1_ESM.docx]

Table S1. Analysis of BMI sensitivity for differences in liver enzymes according to tertiles of urinary phthalate levels.

|  | AST (IU/L) | | | | ALT (IU/L) | | | | γ-GTP (IU/L) | | | |
| --- | --- | --- | --- | --- | --- | --- | --- | --- | --- | --- | --- | --- |
|  | Lsmeans | SE | *p* | *p_trend_* | Lsmeans | SE | *p* | *p_trend_* | Lsmeans | SE | *p* | *p_trend_* |
| 3-5 years of age | | | | | | | | | | | | |
| MBzP | (n=103) |  |  |  | (n=101) |  |  |  | (n=102) |  |  |  |
| T1 | 16.86 | 1.42 | 0.097 | 0.060 | 10.60 | 1.46 | 0.339 | 0.128 | 13.06 | 1.74 | 0.110 | 0.160 |
| T2 | 16.77 | 1.31 |  |  | 11.22 | 1.34 |  |  | 11.96 | 1.62 |  |  |
| T3 | 19.20 | 1.36 |  |  | 12.49 | 1.40 |  |  | 15.19 | 1.67 |  |  |
| MECPP | (n=104) |  |  |  | (n=102) |  |  |  | (n=103) |  |  |  |
| T1 | 16.69 | 1.49 | 0.088 | 0.049 | 9.19 | 1.48 | 0.028 | 0.009 | 11.64 | 1.87 | 0.388 | 0.188 |
| T2 | 16.55 | 1.32 |  |  | 11.18 | 1.31 |  |  | 13.51 | 1.64 |  |  |
| T3 | 19.02 | 1.31 |  |  | 12.63 | 1.30 |  |  | 13.67 | 1.63 |  |  |
| MEHHP | (n=104) |  |  |  | (n=102) |  |  |  | (n=103) |  |  |  |
| T1 | 18.38 | 1.50 | 0.227 | 0.867 | 11.18 | 1.52 | 0.196 | 0.202 | 11.92 | 1.84 | 0.185 | 0.075 |
| T2 | 16.50 | 1.33 |  |  | 10.41 | 1.33 |  |  | 12.47 | 1.62 |  |  |
| T3 | 18.41 | 1.32 |  |  | 12.67 | 1.33 |  |  | 14.64 | 1.61 |  |  |
| MEHP | (n=104) |  |  |  | (n=102) |  |  |  | (n=103) |  |  |  |
| T1 | 18.74 | 1.50 | 0.054 | 0.871 | 12.19 | 1.54 | 0.384 | 0.982 | 15.77 | 1.84 | 0.065 | 0.170 |
| T2 | 15.94 | 1.36 |  |  | 10.51 | 1.39 |  |  | 12.01 | 1.67 |  |  |
| T3 | 18.39 | 1.26 |  |  | 11.92 | 1.29 |  |  | 13.39 | 1.54 |  |  |
| MEOHP | (n=104) |  |  |  | (n=102) |  |  |  | (n=103) |  |  |  |
| T1 | 17.38 | 1.48 | 0.141 | 0.244 | 10.94 | 1.51 | 0.231 | 0.192 | 12.07 | 1.85 | 0.449 | 0.196 |
| T2 | 16.25 | 1.36 |  |  | 10.37 | 1.39 |  |  | 12.90 | 1.69 |  |  |
| T3 | 18.80 | 1.30 |  |  | 12.54 | 1.31 |  |  | 14.10 | 1.61 |  |  |
| MEP | (n=104) |  |  |  | (n=102) |  |  |  | (n=103) |  |  |  |
| T1 | 17.00 | 1.43 | 0.681 | 0.330 | 10.40 | 1.43 | 0.408 | 0.171 | 12.05 | 1.74 | 0.408 | 0.152 |
| T2 | 17.39 | 1.42 |  |  | 11.53 | 1.43 |  |  | 13.07 | 1.73 |  |  |
| T3 | 18.13 | 1.33 |  |  | 12.12 | 1.32 |  |  | 14.21 | 1.62 |  |  |
| MiBP | (n=104) |  |  |  | (n=102) |  |  |  | (n=103) |  |  |  |
| T1 | 18.13 | 1.48 | 0.068 | 0.190 | 10.89 | 1.49 | 0.081 | 0.056 | 12.57 | 1.81 | 0.041 | 0.024 |
| T2 | 16.71 | 1.23 |  |  | 10.98 | 1.24 |  |  | 12.57 | 1.50 |  |  |
| T3 | 19.72 | 1.46 |  |  | 13.46 | 1.47 |  |  | 15.98 | 1.77 |  |  |
| MiNP | (n=104) |  |  |  | (n=102) |  |  |  | (n=103) |  |  |  |
| T1 | 16.68 | 1.42 | 0.496 | 0.356 | 10.93 | 1.44 | 0.798 | 0.566 | 13.37 | 1.76 | 0.956 | 0.857 |
| T2 | 18.12 | 1.41 |  |  | 11.72 | 1.43 |  |  | 13.55 | 1.73 |  |  |
| T3 | 17.84 | 1.34 |  |  | 11.65 | 1.35 |  |  | 13.07 | 1.64 |  |  |
| MnBP | (n=104) |  |  |  | (n=102) |  |  |  | (n=103) |  |  |  |
| T1 | 18.03 | 1.45 | 0.099 | 0.348 | 11.04 | 1.46 | 0.184 | 0.135 | 12.52 | 1.74 | 0.022 | 0.026 |
| T2 | 16.43 | 1.29 |  |  | 10.76 | 1.30 |  |  | 11.98 | 1.55 |  |  |
| T3 | 19.15 | 1.39 |  |  | 12.98 | 1.41 |  |  | 15.95 | 1.67 |  |  |
| ∑DEHP | (n=104) |  |  |  | (n=102) |  |  |  | (n=103) |  |  |  |
| T1 | 17.74 | 1.47 | 0.048 | 0.293 | 11.26 | 1.46 | 0.008 | 0.102 | 12.29 | 1.83 | 0.164 | 0.110 |
| T2 | 15.70 | 1.38 |  |  | 9.13 | 1.36 |  |  | 11.86 | 1.71 |  |  |
| T3 | 18.86 | 1.27 |  |  | 13.18 | 1.25 |  |  | 14.63 | 1.57 |  |  |
| ∑DBP | (n=104) |  |  |  | (n=102) |  |  |  | (n=103) |  |  |  |
| T1 | 18.27 | 1.51 | 0.130 | 0.359 | 11.11 | 1.52 | 0.134 | 0.095 | 13.17 | 1.81 | 0.017 | 0.033 |
| T2 | 16.82 | 1.22 |  |  | 10.95 | 1.23 |  |  | 12.23 | 1.47 |  |  |
| T3 | 19.48 | 1.48 |  |  | 13.37 | 1.50 |  |  | 16.56 | 1.77 |  |  |

MBzP (mono-benzyl phthalate), MECPP [mono(2-ethyl-5-carboxypentyl) phthalate], MEHHP [mono(2-ethyl-5hydroxyhexyl) phthalate], MEHP [mono(2-ethylhexyl) phthalate], MEOHP [mono(2-ethyl-5-oxohexyl) phthalate], MEP (monoethyl phthalate), MiBP (monoisobutyl phthalate), MiNP (monoisononyl phthalate), MnBP (mono-n-butyl phthalate), ΣDEHP [Di(2-ethylhexyl) phthalate] = MECPP, MEHHP, MEHP and MEOHP, ∑DBP (di-n-butyl phthalate) = MiBP and MnBP

AST; aspartate aminotransferase, ALT; alanine aminotransferase, γ-GTP; gamma-glutamyl transferase

Tertiles of Phthalate : MBzP(μg/g creatinine) : T1 : ≤ 7.26 ; T3 : > 16.80 ; MECPP(μg/g creatinine) : T1 : ≤ 87.97 ; T3 : > 152.11 ; MEHHP(μg/g creatinine) : T1 : ≤ 98.76 ; T3 : > 188.49 ; MEHP(μg/g creatinine) : T1 : ≤ 17.17 ; T3 : > 31.69 ; MEOHP(μg/g creatinine) : T1 : ≤ 63.56 ; T3 : > 113.67 ; MEP(μg/g creatinine) : T1 : ≤ 16.81 ; T3 : > 36.02 ; MiBP(μg/g creatinine) : T1 : ≤ 65.73 ; T3 : > 121.24 ; MiNP(μg/g creatinine) : T1 : ≤ 3.94 ; T3 : > 6.36 ; MnBP(μg/g creatinine) : T1 : ≤ 102.16 ; T3 : > 182.81 ; ∑DEHP(μmol/g creatinine) : T1 : ≤ 0.97 ; T3 : > 1.60 ; ∑DBP(μmol/g creatinine) : T1 : ≤ 0.77 ; T3 : > 1.35

* Adjusted for age, sex, mother’s education level, monthly household income at 3–5 years of age, second-hand smoke exposure, and physical inactivity level at 10–15 years of age.

Table S1. Analysis of BMI sensitivity for differences in liver enzymes according to tertiles of urinary phthalate levels (continue).

|  | AST (IU/L) | | | | ALT (IU/L) | | | | γ-GTP (IU/L) | | | | |
| --- | --- | --- | --- | --- | --- | --- | --- | --- | --- | --- | --- | --- | --- |
|  | Lsmeans | SE | *p* | *p_trend_* | Lsmeans | SE | *p* | *p_trend_* | Lsmeans | | SE | *p* | *p_trend_* |
| 7-9 years of age | | | | | | | | | | | | | |
| MBzP | (n=111) |  |  |  | (n=109) |  |  |  | (n=110) |  | |  |  |
| T1 | 17.39 | 1.34 | 0.921 | 0.605 | 11.78 | 1.37 | 0.865 | 0.981 | 13.37 | 1.72 | | 0.742 | 0.532 |
| T2 | 17.44 | 1.24 |  |  | 11.12 | 1.25 |  |  | 13.07 | 1.58 | |  |  |
| T3 | 17.84 | 1.31 |  |  | 11.73 | 1.34 |  |  | 14.26 | 1.68 | |  |  |
| MECPP | (n=111) |  |  |  | (n=109) |  |  |  | (n=110) |  | |  |  |
| T1 | 17.03 | 1.37 | 0.245 | 0.114 | 10.20 | 1.40 | 0.289 | 0.135 | 11.16 | 1.75 | | 0.120 | 0.176 |
| T2 | 17.06 | 1.18 |  |  | 11.60 | 1.20 |  |  | 14.36 | 1.50 | |  |  |
| T3 | 18.80 | 1.29 |  |  | 12.22 | 1.32 |  |  | 13.85 | 1.64 | |  |  |
| MEHHP | (n=111) |  |  |  | (n=109) |  |  |  | (n=110) |  | |  |  |
| T1 | 17.20 | 1.31 | 0.024 | 0.045 | 10.82 | 1.37 | 0.364 | 0.194 | 11.94 | 1.72 | | 0.292 | 0.151 |
| T2 | 16.34 | 1.17 |  |  | 11.07 | 1.22 |  |  | 13.65 | 1.53 | |  |  |
| T3 | 19.47 | 1.24 |  |  | 12.46 | 1.29 |  |  | 14.36 | 1.63 | |  |  |
| MEHP | (n=111) |  |  |  | (n=109) |  |  |  | (n=110) |  | |  |  |
| T1 | 17.12 | 1.44 | 0.637 | 0.296 | 10.53 | 1.46 | 0.510 | 0.253 | 11.18 | 1.82 | | 0.163 | 0.133 |
| T2 | 17.39 | 1.14 |  |  | 11.49 | 1.15 |  |  | 13.99 | 1.44 | |  |  |
| T3 | 18.26 | 1.38 |  |  | 12.00 | 1.40 |  |  | 13.75 | 1.74 | |  |  |
| MEOHP | (n=111) |  |  |  | (n=109) |  |  |  | (n=110) |  | |  |  |
| T1 | 16.52 | 1.34 | 0.196 | 0.059 | 10.44 | 1.37 | 0.432 | 0.211 | 11.79 | 1.72 | | 0.204 | 0.444 |
| T2 | 17.25 | 1.17 |  |  | 11.55 | 1.20 |  |  | 14.52 | 1.51 | |  |  |
| T3 | 18.74 | 1.28 |  |  | 12.10 | 1.31 |  |  | 13.18 | 1.64 | |  |  |
| MEP | (n=111) |  |  |  | (n=109) |  |  |  | (n=110) |  | |  |  |
| T1 | 16.86 | 1.23 | 0.089 | 0.106 | 10.71 | 1.26 | 0.222 | 0.146 | 12.64 | 1.61 | | 0.602 | 0.321 |
| T2 | 16.12 | 1.36 |  |  | 10.55 | 1.39 |  |  | 13.84 | 1.77 | |  |  |
| T3 | 18.71 | 1.20 |  |  | 12.50 | 1.23 |  |  | 14.15 | 1.57 | |  |  |
| MiBP | (n=111) |  |  |  | (n=109) |  |  |  | (n=110) |  | |  |  |
| T1 | 15.31 | 1.22 | 0.005 | 0.001 | 9.93 | 1.28 | 0.108 | 0.055 | 12.71 | 1.63 | | 0.305 | 0.815 |
| T2 | 18.22 | 1.32 |  |  | 12.23 | 1.39 |  |  | 15.23 | 1.76 | |  |  |
| T3 | 19.14 | 1.17 |  |  | 12.43 | 1.22 |  |  | 13.32 | 1.56 | |  |  |
| MiNP | (n=111) |  |  |  | (n=109) |  |  |  | (n=110) |  | |  |  |
| T1 | 16.60 | 1.29 | 0.345 | 0.281 | 10.95 | 1.31 | 0.655 | 0.389 | 11.65 | 1.62 | | 0.065 | 0.173 |
| T2 | 18.35 | 1.31 |  |  | 11.13 | 1.35 |  |  | 15.26 | 1.65 | |  |  |
| T3 | 17.80 | 1.21 |  |  | 12.03 | 1.24 |  |  | 13.90 | 1.53 | |  |  |
| MnBP | (n=111) |  |  |  | (n=109) |  |  |  | (n=110) |  | |  |  |
| T1 | 16.40 | 1.25 | 0.047 | 0.009 | 10.53 | 1.28 | 0.089 | 0.040 | 12.38 | 1.64 | | 0.469 | 0.454 |
| T2 | 17.21 | 1.24 |  |  | 11.04 | 1.27 |  |  | 14.36 | 1.62 | |  |  |
| T3 | 19.24 | 1.24 |  |  | 13.04 | 1.27 |  |  | 13.79 | 1.63 | |  |  |
| ∑DEHP | (n=111) |  |  |  | (n=109) |  |  |  | (n=110) |  | |  |  |
| T1 | 17.17 | 1.39 | 0.516 | 0.233 | 10.42 | 1.42 | 0.455 | 0.254 | 11.23 | 1.76 | | 0.112 | 0.530 |
| T2 | 17.40 | 1.14 |  |  | 11.69 | 1.16 |  |  | 14.55 | 1.43 | |  |  |
| T3 | 18.52 | 1.39 |  |  | 11.98 | 1.41 |  |  | 12.57 | 1.76 | |  |  |
| ∑DBP | (n=111) |  |  |  | (n=109) |  |  |  | (n=110) |  | |  |  |
| T1 | 15.99 | 1.26 | 0.003 | 0.001 | 10.31 | 1.30 | 0.013 | 0.006 | 12.32 | 1.70 | | 0.515 | 0.332 |
| T2 | 16.92 | 1.17 |  |  | 10.72 | 1.21 |  |  | 13.98 | 1.58 | |  |  |
| T3 | 19.69 | 1.20 |  |  | 13.44 | 1.23 |  |  | 13.91 | 1.61 | |  |  |

MBzP (mono-benzyl phthalate), MECPP [mono(2-ethyl-5-carboxypentyl) phthalate], MEHHP [mono(2-ethyl-5hydroxyhexyl) phthalate], MEHP [mono(2-ethylhexyl) phthalate], MEOHP [mono(2-ethyl-5-oxohexyl) phthalate], MEP (monoethyl phthalate), MiBP (monoisobutyl phthalate), MiNP (monoisononyl phthalate), MnBP (mono-n-butyl phthalate), ΣDEHP [Di(2-ethylhexyl) phthalate] = MECPP, MEHHP, MEHP and MEOHP, ∑DBP (di-n-butyl phthalate) = MiBP and MnBP

AST; aspartate aminotransferase, ALT; alanine aminotransferase, γ-GTP; gamma-glutamyl transferase

Tertiles of Phthalate : MBzP(μg/g creatinine) : T1 : ≤ 5.97 ; T3 : > 13.19 ; MECPP(μg/g creatinine) : T1 : ≤ 55.91 ; T3 : > 100.57 ; MEHHP(μg/g creatinine) : T1 : ≤ 69.00 ; T3 : > 110.70 ; MEHP(μg/g creatinine) : T1 : ≤ 12.93 ; T3 : > 24.57 ; MEOHP(μg/g creatinine) : T1 : ≤ 39.49 ; T3 : > 66.81 ; MEP(μg/g creatinine) : T1 : ≤ 10.35 ; T3 : > 18.86 ; MiBP(μg/g creatinine) : T1 : ≤ 49.64 ; T3 : > 81.93 ; MiNP(μg/g creatinine) : T1 : ≤ 3.41 ; T3 : > 5.65 ; MnBP(μg/g creatinine) : T1 : ≤ 76.64 ; T3 : > 134.57 ; ∑DEHP(μmol/g creatinine) : T1 : ≤ 0.64 ; T3 : > 1.04 ; ∑DBP(μmol/g creatinine) : T1 : ≤ 0.55 ; T3 : > 0.99

* Adjusted for age, sex, mother’s education level, monthly household income at 7-9 years of age, second-hand smoke exposure, and physical inactivity level at 10–15 years of age.

Figure S1. A directed acyclic graph (DAG) depicting the causal relationship between exposure to phthalate metabolites and liver function.


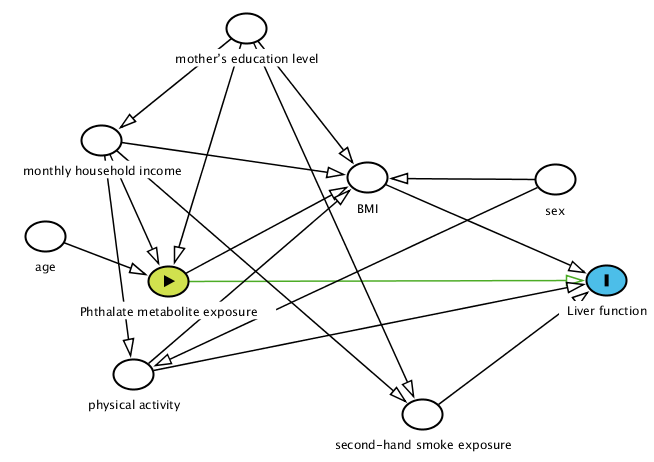


Figure S2. Non-linear relationship of aspartate aminotransferase(AST) with urinary phthalate levels at 3-5 years of age.


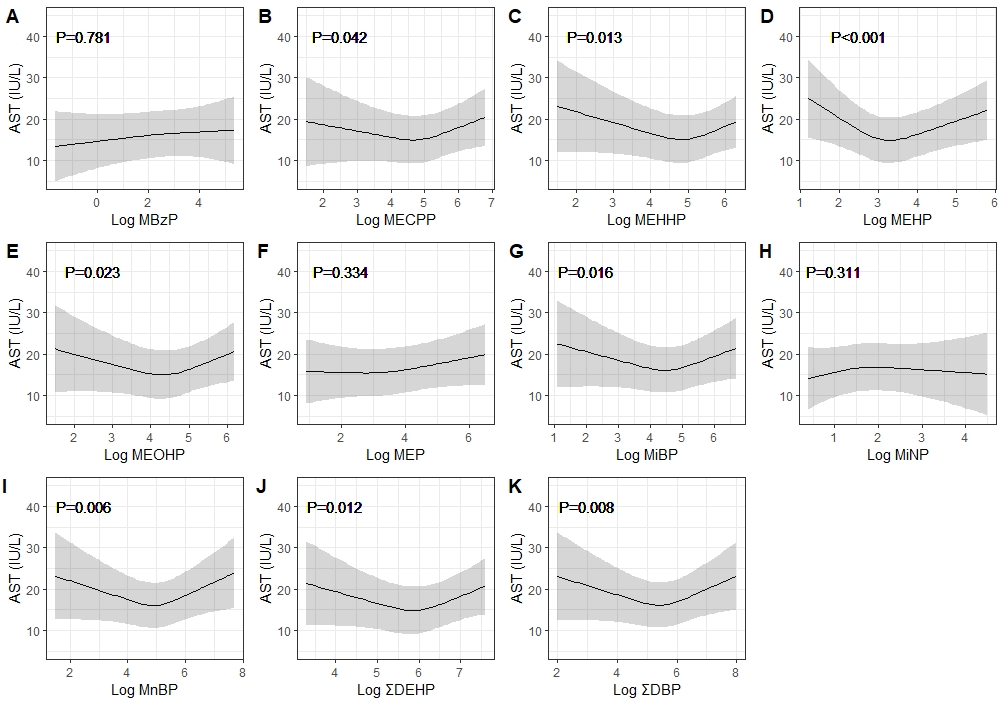


(A) MBzP (mono-benzyl phthalate), (B) MECPP [mono(2-ethyl-5-carboxypentyl) phthalate], (C) MEHHP [mono(2-ethyl-5hydroxyhexyl) phthalate], (D) MEHP [mono(2-ethylhexyl) phthalate], (E) MEOHP [mono(2-ethyl-5-oxohexyl) phthalate], (F) MEP (monoethyl phthalate), (G) MiBP (monoisobutyl phthalate), (H) MiNP (monoisononyl phthalate), (I) MnBP (mono-n-butyl phthalate), (J) ΣDEHP [Di(2-ethylhexyl) phthalate] = MECPP, MEHHP, MEHP and MEOHP, (K) ∑DBP (di-n-butyl phthalate) = MiBP and MnBP

AST; aspartate aminotransferase

Non-linear relationships were assessed using restricted cubic spline after adjusting for age, sex, body mass index, mother’s education level, monthly household income at 3–5 years of age, second-hand smoke exposure, and physical inactivity level at 10–15 years of age. P value represent nonlinear test.

Figure S3. Non-linear relationship of alanine aminotransferase(ALT) with urinary phthalate levels at 3-5 years of age.


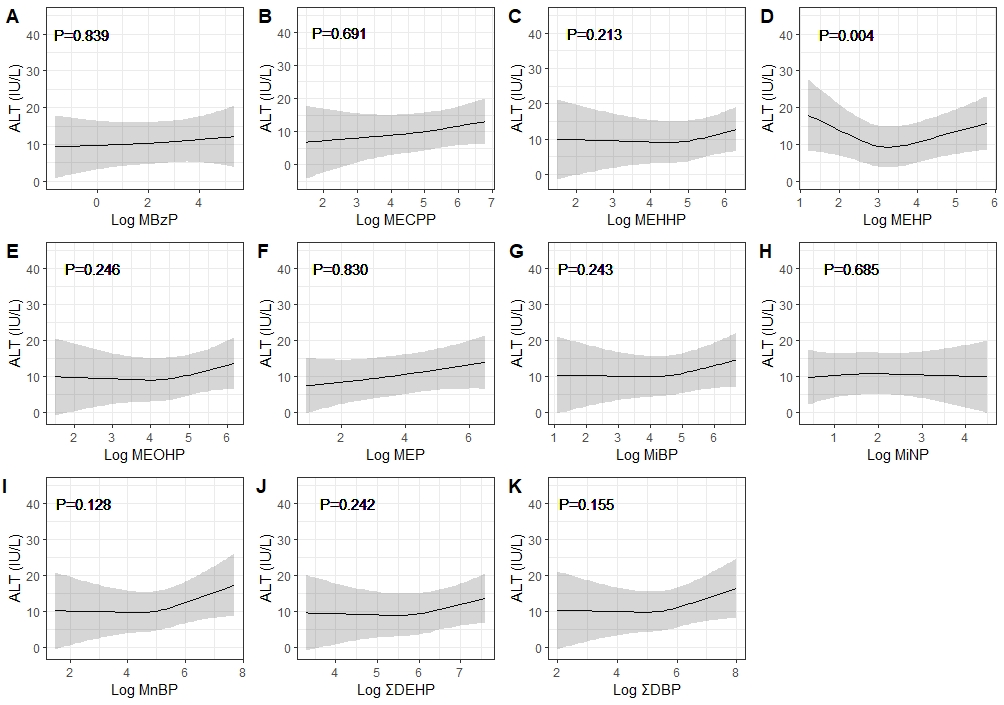


(A) MBzP (mono-benzyl phthalate), (B) MECPP [mono(2-ethyl-5-carboxypentyl) phthalate], (C) MEHHP [mono(2-ethyl-5hydroxyhexyl) phthalate], (D) MEHP [mono(2-ethylhexyl) phthalate], (E) MEOHP [mono(2-ethyl-5-oxohexyl) phthalate], (F) MEP (monoethyl phthalate), (G) MiBP (monoisobutyl phthalate), (H) MiNP (monoisononyl phthalate), (I) MnBP (mono-n-butyl phthalate), (J) ΣDEHP [Di(2-ethylhexyl) phthalate] = MECPP, MEHHP, MEHP and MEOHP, (K) ∑DBP (di-n-butyl phthalate) = MiBP and MnBP

ALT; alanine aminotransferase

Non-linear relationships were assessed using restricted cubic spline after adjusting for age, sex, body mass index, mother’s education level, monthly household income at 3–5 years of age, second-hand smoke exposure, and physical inactivity level at 10–15 years of age. P value represent nonlinear test.

Figure S4. Non-linear relationship of gamma-glutamyl transferase(γ-GTP) with urinary phthalate levels at 3-5 years of age.


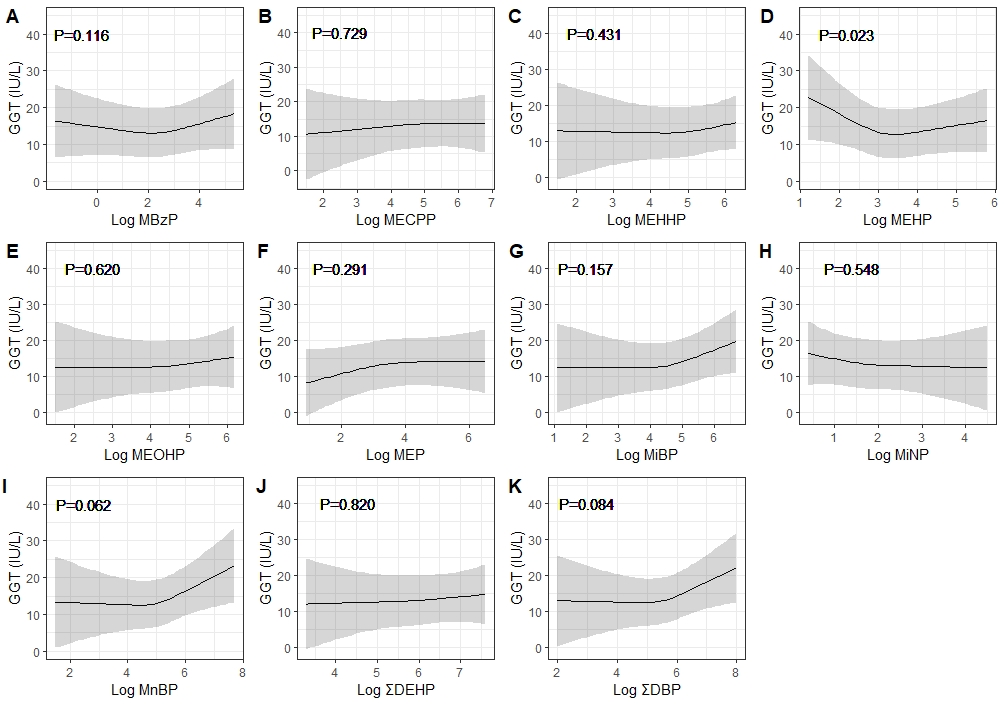


(A) MBzP (mono-benzyl phthalate), (B) MECPP [mono(2-ethyl-5-carboxypentyl) phthalate], (C) MEHHP [mono(2-ethyl-5hydroxyhexyl) phthalate], (D) MEHP [mono(2-ethylhexyl) phthalate], (E) MEOHP [mono(2-ethyl-5-oxohexyl) phthalate], (F) MEP (monoethyl phthalate), (G) MiBP (monoisobutyl phthalate), (H) MiNP (monoisononyl phthalate), (I) MnBP (mono-n-butyl phthalate), (J) ΣDEHP [Di(2-ethylhexyl) phthalate] = MECPP, MEHHP, MEHP and MEOHP, (K) ∑DBP (di-n-butyl phthalate) = MiBP and MnBP

γ-GTP ; gamma-glutamyl transferase

Non-linear relationships were assessed using restricted cubic spline after adjusting for age, sex, body mass index, mother’s education level, monthly household income at 3–5 years of age, second-hand smoke exposure, and physical inactivity level at 10–15 years of age. P value represent nonlinear test.

Figure S5. Non-linear relationship of aspartate aminotransferase(AST) with urinary phthalate levels at 7-9 years of age.


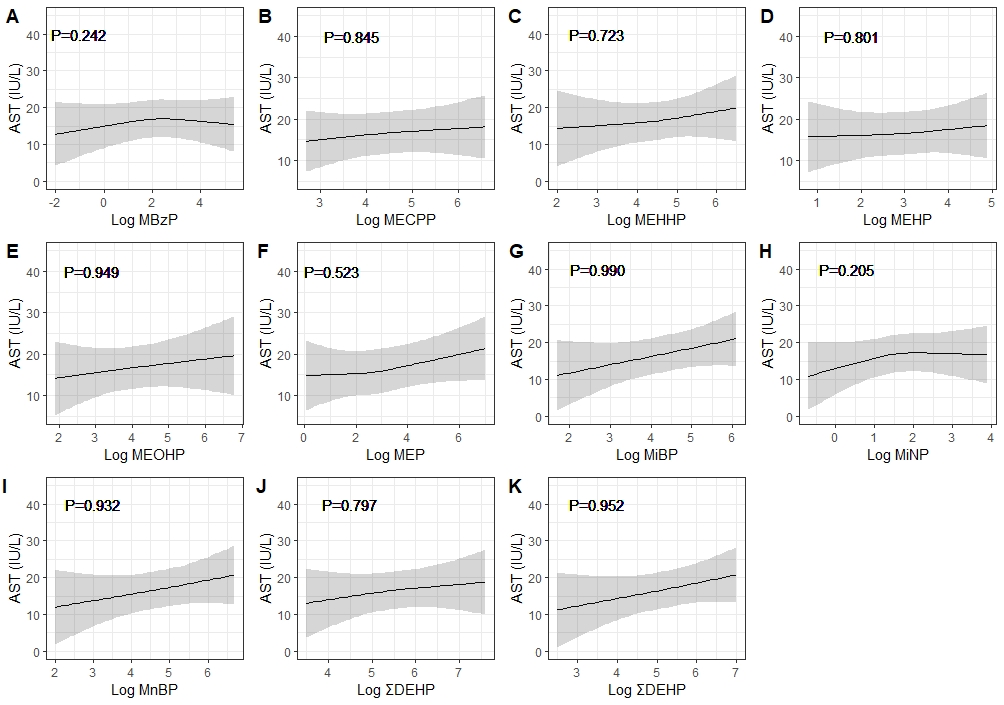


(A) MBzP (mono-benzyl phthalate), (B) MECPP [mono(2-ethyl-5-carboxypentyl) phthalate], (C) MEHHP [mono(2-ethyl-5hydroxyhexyl) phthalate], (D) MEHP [mono(2-ethylhexyl) phthalate], (E) MEOHP [mono(2-ethyl-5-oxohexyl) phthalate], (F) MEP (monoethyl phthalate), (G) MiBP (monoisobutyl phthalate), (H) MiNP (monoisononyl phthalate), (I) MnBP (mono-n-butyl phthalate), (J) ΣDEHP [Di(2-ethylhexyl) phthalate] = MECPP, MEHHP, MEHP and MEOHP, (K) ∑DBP (di-n-butyl phthalate) = MiBP and MnBP

AST; aspartate aminotransferase

Non-linear relationships were assessed using restricted cubic spline after adjusting for age, sex, body mass index, mother’s education level, monthly household income at 7–9 years of age, second-hand smoke exposure, and physical inactivity level at 10–15 years of age. P value represent nonlinear test.

Figure S6. Non-linear relationship of alanine aminotransferase(ALT) with urinary phthalate levels at 7-9 years of age.


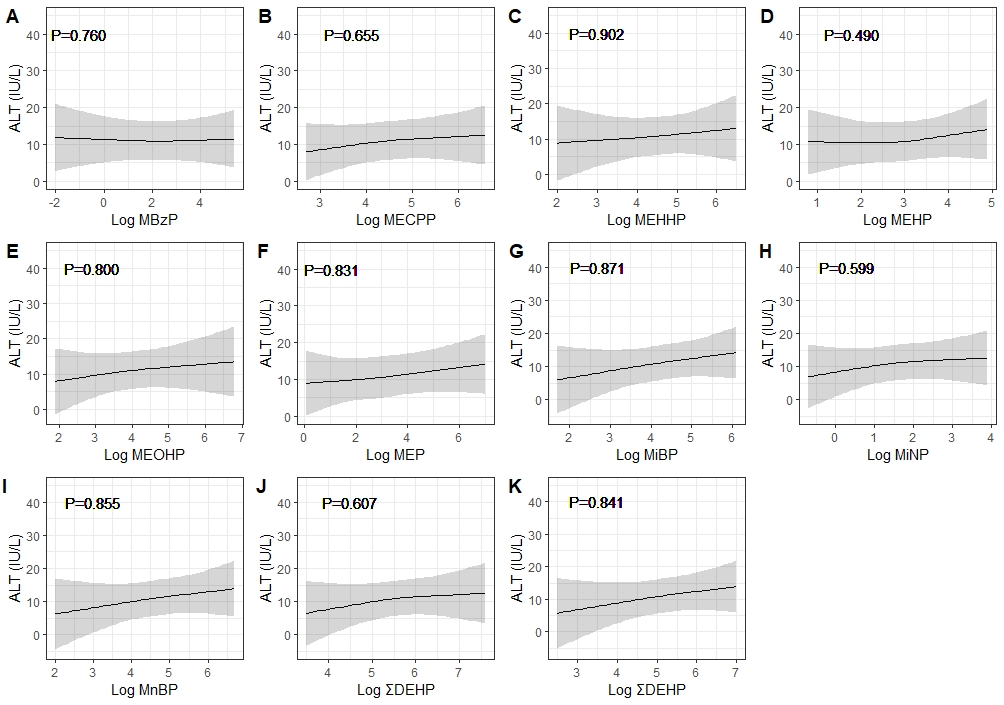


(A) MBzP (mono-benzyl phthalate), (B) MECPP [mono(2-ethyl-5-carboxypentyl) phthalate], (C) MEHHP [mono(2-ethyl-5hydroxyhexyl) phthalate], (D) MEHP [mono(2-ethylhexyl) phthalate], (E) MEOHP [mono(2-ethyl-5-oxohexyl) phthalate], (F) MEP (monoethyl phthalate), (G) MiBP (monoisobutyl phthalate), (H) MiNP (monoisononyl phthalate), (I) MnBP (mono-n-butyl phthalate), (J) ΣDEHP [Di(2-ethylhexyl) phthalate] = MECPP, MEHHP, MEHP and MEOHP, (K) ∑DBP (di-n-butyl phthalate) = MiBP and MnBP

ALT; alanine aminotransferase

Non-linear relationships were assessed using restricted cubic spline after adjusting for age, sex, body mass index, mother’s education level, monthly household income at 7–9 years of age, second-hand smoke exposure, and physical inactivity level at 10–15 years of age. P value represent nonlinear test.

Figure S7. Non-linear relationship of gamma-glutamyl transferase(γ-GTP) with urinary phthalate levels at 7-9 years of age.


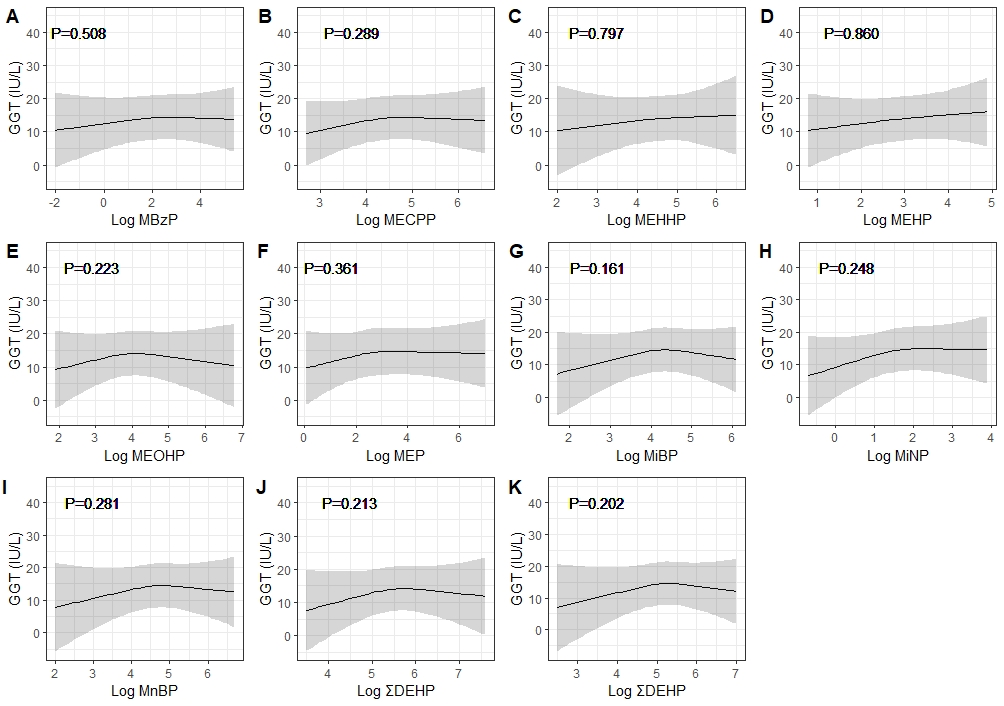


(A) MBzP (mono-benzyl phthalate), (B) MECPP [mono(2-ethyl-5-carboxypentyl) phthalate], (C) MEHHP [mono(2-ethyl-5hydroxyhexyl) phthalate], (D) MEHP [mono(2-ethylhexyl) phthalate], (E) MEOHP [mono(2-ethyl-5-oxohexyl) phthalate], (F) MEP (monoethyl phthalate), (G) MiBP (monoisobutyl phthalate), (H) MiNP (monoisononyl phthalate), (I) MnBP (mono-n-butyl phthalate), (J) ΣDEHP [Di(2-ethylhexyl) phthalate] = MECPP, MEHHP, MEHP and MEOHP, (K) ∑DBP (di-n-butyl phthalate) = MiBP and MnBP

γ-GTP ; gamma-glutamyl transferase

Non-linear relationships were assessed using restricted cubic spline after adjusting for age, sex, body mass index, mother’s education level, monthly household income at 7–9 years of age, second-hand smoke exposure, and physical inactivity level at 10–15 years of age. P value represent nonlinear test.
